# Refractory Obstructive Sleep Apnea in a Patient with Diffuse Idiopathic Skeletal Hyperostosis

**DOI:** 10.1155/2016/4906863

**Published:** 2016-11-09

**Authors:** Ara Darakjian, Ani B. Darakjian, Edward T. Chang, Macario Camacho

**Affiliations:** ^1^Department of Psychiatry and Behavioral Sciences, Keck School of Medicine, 1975 Zonal Ave, Los Angeles, CA 90033, USA; ^2^Department of Radiology, Southern California Permanente Medical Group, 4867 W. Sunset Blvd, Los Angeles, CA 90027, USA; ^3^Division of Otolaryngology-Head and Neck Surgery, Tripler Army Medical Center, 1 Jarrett White Rd, Honolulu, HI 96859, USA; ^4^Department of Psychiatry and Behavioral Sciences, Sleep Medicine Division, Stanford Hospital and Clinics, 450 Broadway St, Pavillion B., Redwood City, CA 94063, USA

## Abstract

Diffuse Idiopathic Skeletal Hyperostosis (DISH) can cause ossification of ligaments and may affect the spine. We report a case of obstructive sleep apnea in a patient with significant upper airway narrowing secondary to cervical DISH. This patient had an initial apnea-hypopnea index (AHI) of 145 events/hour and was treated with uvulopalatopharyngoplasty, genial tubercle advancement, hyoid suspension, septoplasty, inferior turbinoplasties, and radiofrequency ablations to the tongue base which reduced his AHI to 40 events/hour. He redeveloped symptoms, was started on positive airway pressure (PAP) therapy, and later underwent a maxillomandibular advancement which improved his AHI to 16.3 events/hour. A few years later his AHI was 100.4 events/hour. His disease has gradually progressed over time and he was restarted on PAP therapy. Despite PAP titration, years of using PAP therapy, and being 100 percent compliant for the past three months (average daily use of 7.6 hours/night), he has an AHI of 5.1 events/hour and has persistent hypersomnia with an Epworth Sleep Scale questionnaire score of 18/24. At this time he is pending further hypersomnia work-up. DISH patients require prolonged follow-up to monitor the progression of disease, and they may require unconventional measures for adequate treatment of obstructive sleep apnea.

## 1. Introduction

Diffuse Idiopathic Skeletal Hyperostosis (DISH) is an idiopathic noninflammatory disease that is characterized by calcification and ossification of spinal ligaments and enthuses [1]. It is a surprisingly common condition, being found in 6–12% of a population that underwent an autopsy for other reasons [1]. DISH can be asymptomatic; however, in those who are symptomatic, the most common manifestations include a decreased range of motion in the thoracic spine, shoulder pain, and neck pain. Most cases are sufficiently managed with NSAIDs and supportive therapies. Less commonly, DISH has been reported to cause dysphagia and obstructive sleep apnea secondary to narrowing of the upper airway [2], but few cases are severe enough to manifest in this way. Previous reports in the literature of obstructive sleep apnea secondary to DISH have shown successful treatment with continuous positive airway pressure (CPAP) therapy [2–4]. We describe the complicated treatment course of a patient with DISH, whose large anterior cervical osteophytes caused significant narrowing of his upper airway and subsequent sleep apnea. While sleep surgeries initially led to resolution of symptoms, the patient continued to have an elevated apnea-hypopnea index and required further surgery as well as positive airway pressure therapy due to recurrence of obstructive sleep apnea.

## 2. Case Presentation

A 38-year-old man presented to the sleep medicine clinic with hypersomnia. A polysomnogram demonstrated obstructive sleep apnea (OSA) with an apnea-hypopnea index (AHI) of 145 events/hour. The patient was treated with CPAP therapy at 9 cm of water pressure (cwp). He was unable to tolerate CPAP and had significant symptoms, and therefore he was referred to a sleep surgeon for evaluation. The patient underwent sleep surgery in 1996, consisting of uvulopalatopharyngoplasty (UPPP), genial tubercle advancement, hyoid suspension, septoplasty, and bilateral inferior turbinate reduction. The patient had such significant improvement in his symptoms that he did not use CPAP therapy after surgery (however, it is unknown to what extent his AHI improved, if at all, since no sleep study was performed after surgery). Two years after the sleep surgery, the patient subsequently complained of dysphagia, neck stiffness, headache, and tingling in his left hand. After various forms of imaging he was diagnosed with DISH with significant anterior cervical osteophytes at the C5-C6 level.

Over the next three years, he developed hypersomnia and was treated with five radiofrequency ablations of the tongue base. After the treatment, he noted significant improvement in symptoms. However, his neck pain worsened to the point where he required a C5-C6 discectomy. The patient subsequently redeveloped hypersomnia in 2002 and a repeat polysomnogram demonstrated an AHI of 40 events/hour. The patient again attempted CPAP but was intolerant to the therapy and underwent an advancement of the maxillomandibular complex by 10 mm. Despite a reduction of the AHI to 16.3 events/hour, the patient's hypersomnia persisted. The patient was then counseled to reattempt treatment with CPAP. In 2006, the patient underwent a split night study which demonstrated an AHI of 100.4 events/hour and a positive airway pressure titration study recommending bilevel positive airway pressure (bilevel) therapy, set to an inspiratory positive airway pressure (IPAP) of 10 centimeters of water pressure (cwp) and expiratory positive airway pressure (EPAP) of 6 cwp. Over the years his hypersomnia progressively worsened, requiring bilevel titration, and his new pressures were an IPAP of 22 cwp and an EPAP of 14 cwp.

**Figure 1:**
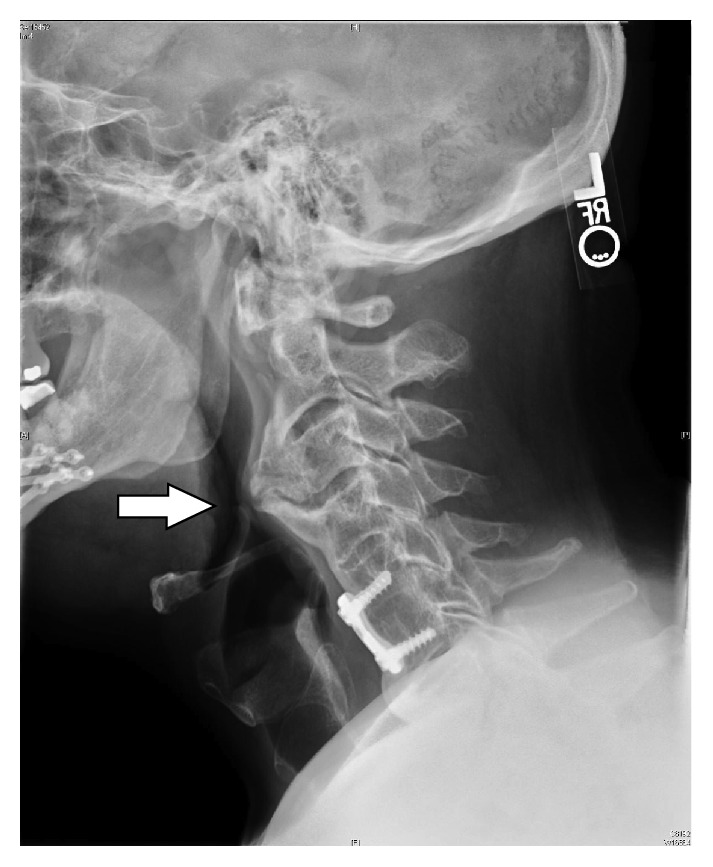
Large anterior osteophytes involving C2–4 (white arrow). Note there is sparing of the disc spaces. The bulge narrows the upper airway, abutting the epiglottis.

Today, the patient is 57 years old and has continued to use positive airway pressure therapy. The bilevel data download demonstrates 100% compliance for the past three months and an average daily use of 7.6 hours/night. Despite the higher pressures, there was a residual device downloaded AHI of 5.1/h and the patient had persistent hypersomnia with an Epworth Sleep Scale (ESS) questionnaire score of 18/24 (≥11 being the cutoff for hypersomnia). Physical examination reveals that the patient is 71 inches and 240 lbs (body mass index of 33.5 kg/m^2^) with a blood pressure of 143/76. His nasal septum is straight and his inferior turbinates are nonobstructing bilaterally (grades 1 or 2) [5]. He has a high-arched and narrow hard palate, an overjet of 3 mm, tongue scalloping, and a Grade 3 Friedman Palate Position [6]. A plain neck radiograph (Figure 1) demonstrates large anterior cervical spine osteophytes involving C2 through C4, with sparing of the disc space and clear obstruction of the upper airway. The osteophytes are so large that they abut against the epiglottis. Potential causes for the patient's hypersomnia that were explored include (1) insomnia secondary to pain where he awakens several times throughout the night secondary to neck pain, currently treated with gabapentin 900 mg three times a day, and sometimes takes hydrocodone when the pain is significant, (2) history of prostate hypertrophy with nocturnal awakenings treated with tamsulosin 0.4 mg daily, (3) higher bilevel pressures awaken him because of “the sensation of strong airflow,” and (4) restless leg syndrome treated with pramipexole 0.5 mg daily. Despite the appropriate medical management, the patient's hypersomnia has persisted, so he was prescribed modafinil 200 mg each morning. The patient states that the modafinil helps somewhat during the day, but it does not help him enough. In order to further assist the patient in reducing his hypersomnia, we are considering the following: (1) a new bilevel titration study to improve his experience with positive airway pressure therapy, (2) a multiple sleep latency test (MSLT) to help rule out narcolepsy, and (3) blood testing for other sources that can cause fatigue, sleepiness, and tiredness to include testosterone levels and iron levels.

## 3. Discussion

This case study patient presents with classic clinical manifestations and radiographic findings of DISH. He describes dysphagia and a stiff neck with a decreased range of motion in addition to neck and shoulder pain. The radiographic findings are consistent with DISH, which is described as “flowing mantles” of ossification most commonly involving the anterior longitudinal ligament, without significant narrowing of the disc space [7, 8]. Given that the upper airway can narrow significantly (33%) in OSA patients by the simple act of changing from the upright to the supine positions [9], it is not surprising that, with such large osteophytes, the patient is experiencing upper airway obstruction during sleep.

OSA is a common disorder, and CPAP is a highly efficacious treatment [10]. However, as in this patient, the effectiveness is limited by compliance, which is estimated to be 46–83% when adherence is defined as greater than 4 hours of nightly use [11]. In addition to CPAP or bilevel, other medical options include mandibular advancement devices, weight loss, positional therapy, and wedge pillows; however, the efficacy and effectiveness of each of these are variable. After exhausting medical management, soft tissue sleep surgery or maxillomandibular advancement surgeries are commonly used to relieve upper airway obstruction. When performing soft tissue sleep surgery, the most effective technique has been demonstrated to be multilevel surgeries that target the sites of obstruction, including nasal surgeries, tongue reduction/stabilization or advancement surgeries, oropharyngeal surgeries, and hypopharyngeal surgeries. A systematic review of multilevel surgeries has demonstrated a 66.4% success rate [12]. Maxillomandibular advancement, one of the most effective surgeries for sleep apnea [13], can be performed either without soft tissue sleep surgery or as a second surgery in patients not successfully treated with soft tissue sleep surgery. In this patient, it is unclear why there was a sixfold increase in the AHI after the maxillomandibular advancement (from 16.3 events/hour to 100.4 events/hour) over a matter of four years.

This patient currently uses a bilevel positive airway pressure (bilevel) machine, which is used for patients that have a persistently elevated AHI despite use of CPAP. According to the 2013 Official American Thoracic Society Statement for CPAP adherence and tracking, an AHI < 10 events/hour is considered effective treatment. Therefore, we consider our patient, with residual AHI of 5.1, to be effectively treated and are considering other sources for his hypersomnia [14]. However, given his persistent hypersomnia and residual AHI, there is an argument for another BiPAP titration study with a goal of further reducing his AHI, especially if other causes for his hypersomnia are ruled out.

## 4. Conclusion

DISH is a common disorder that can cause obstructive sleep apnea secondary to large anterior cervical osteophyte formation in severe cases. This case study demonstrates the need to follow the patients in the long term as they may initially do well with soft tissue sleep surgery or maxillomandibular advancement surgeries but may require positive airway pressure via CPAP or bilevel in the long term.
